# The perfect ileal pouch–anal anastomosis

**DOI:** 10.1007/s10151-022-02700-2

**Published:** 2022-09-17

**Authors:** N. Ecker, A.-C. Woywod, K.-W. Ecker

**Affiliations:** 1 Forstmeisterweg 65, 23564 Lübeck, Germany; 2 “Dr. Nölke,” Königstraße 22, 25348 Glückstadt, Germany; 3 Department of General, Visceral, Vascular, and Pediatric Surgery, University of Saarland, Homburg, Saar Germany; 4 Emeritus Director of the Surgical Department, MediClin Müritz-Klinikum, Weinbergstraße 19, 17192 Waren, Germany; 5 Tannenweg 1, 22889 Tangstedt, Germany

In ileal pouch-anal anastomosis, pitfalls may result from inappropriate anastomotic height, rectal cuff, sphincter damage, residual rectal mucosa, dog-ears in double-stapler technique (persistent inflammation, risk of malignancy), and temporary fecal diversion. Most surgical techniques either overvalue or neglect particular aspects (Figs. 1, 2, 3, 4, 5, 6, 7, 8, 9).

**Fig. 1:**
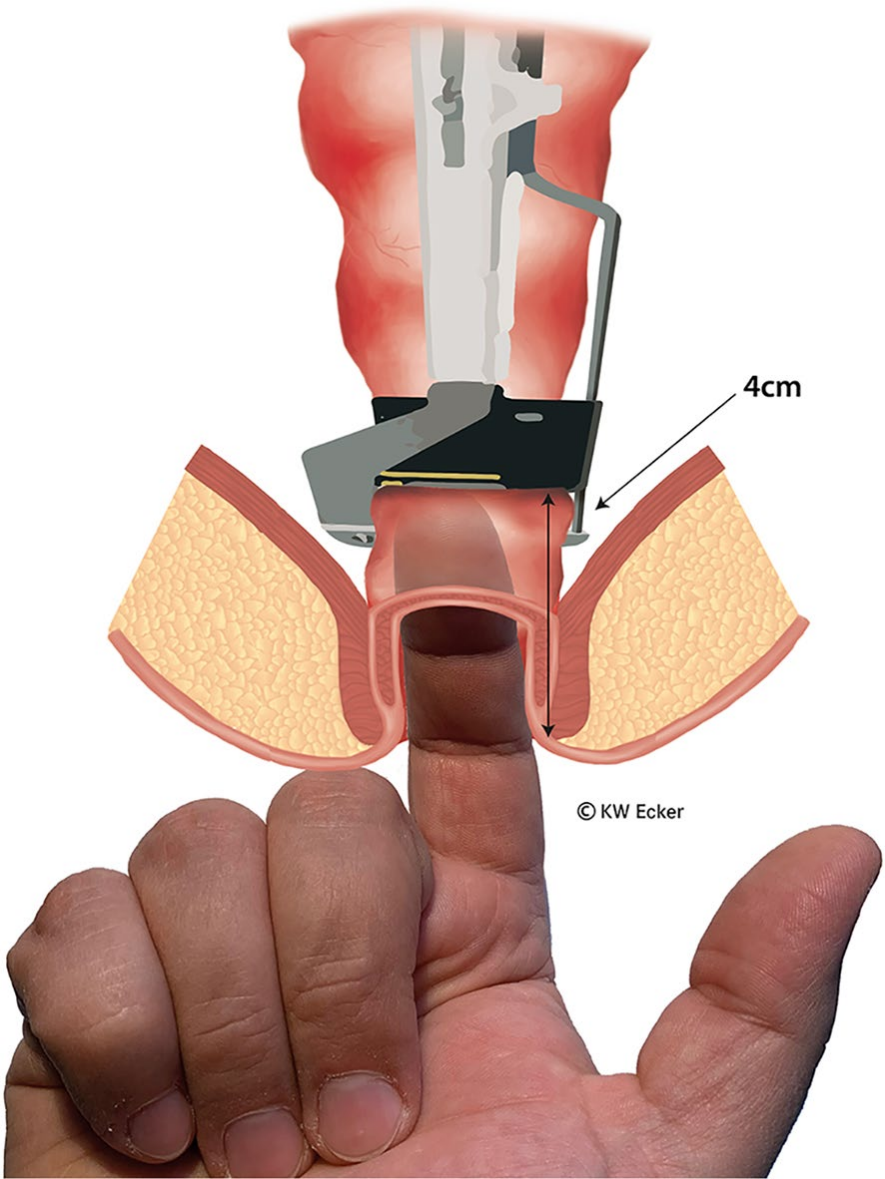
**Determination of the height of the rectal resection**. The rectum is ideally resected at a height identified when the tip of the index finger is inserted transanally up to the second flexural crease and just feels the 30 mm linear stapler. This height of approximately 4 cm from the anocutaneous junction will be reduced by the subsequent steps

**Fig. 2:**
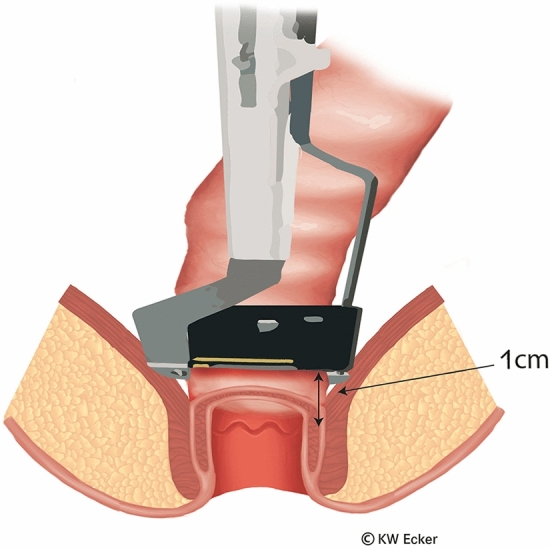
**Definitive positioning of the linear stapler.** After moving the stapler slightly distally, it is closed and fired so that the rectum can be easily resected with the scalpel above the closed device. The staple suture line is then approximately 1 cm above the dentate line

**Fig. 3:**
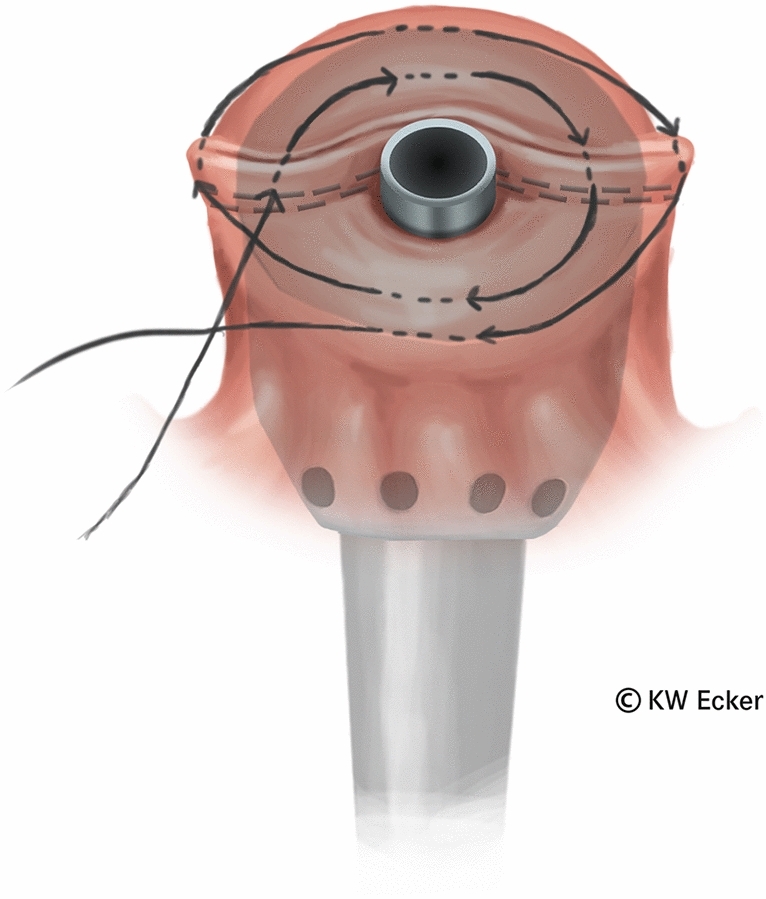
**Stitching a modified Asao suture.** After the head of the largest possible (28–32 mm) circular stapler has been precisely positioned transanally, a purse-string suture is stitched in a double spiral. In this process, the lateral ends of the staple suture (dogears) are deeply and securely incorporated in the outer helix

**Fig. 4:**
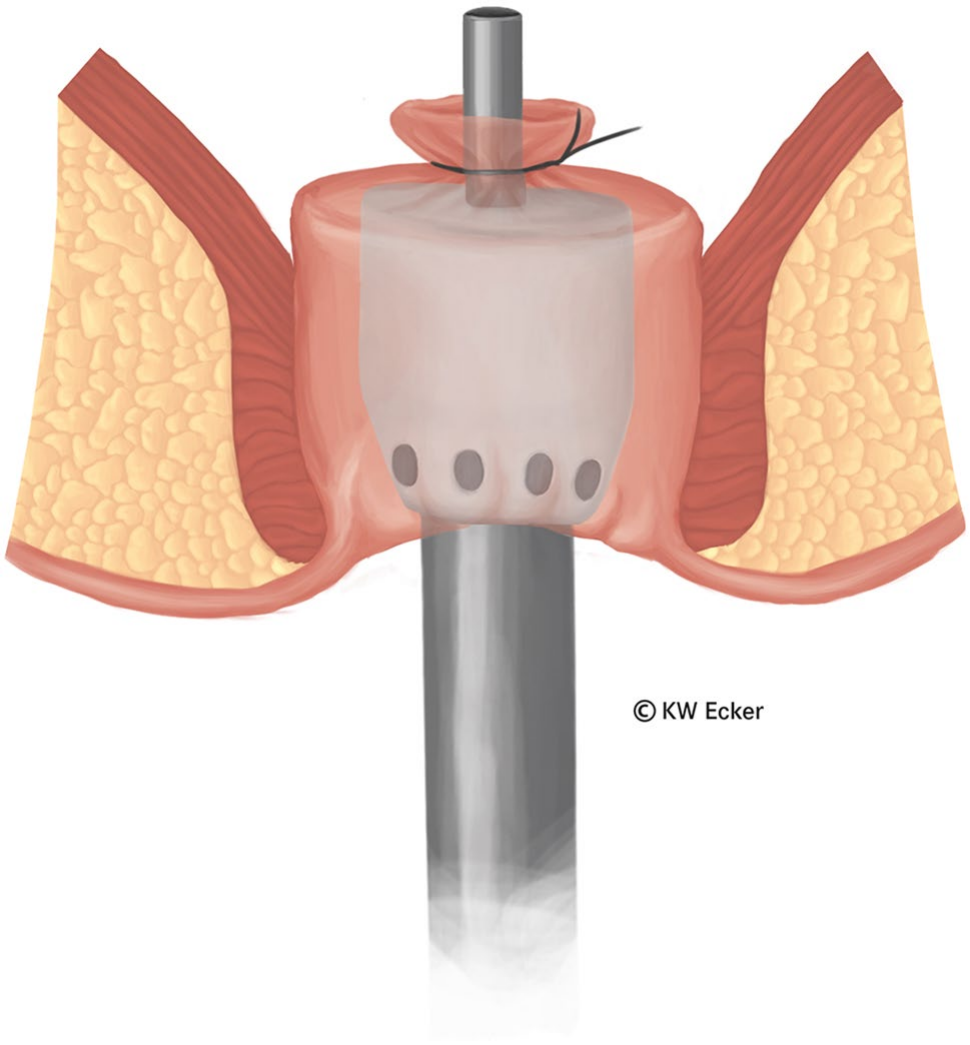
**Knotting the modified Asao suture.** After the suture material has been carefully tightened, the ends are knotted. This incorporates the whole linear stapler line as well as all excess tissue centrally around the guide mandrel

**Fig. 5:**
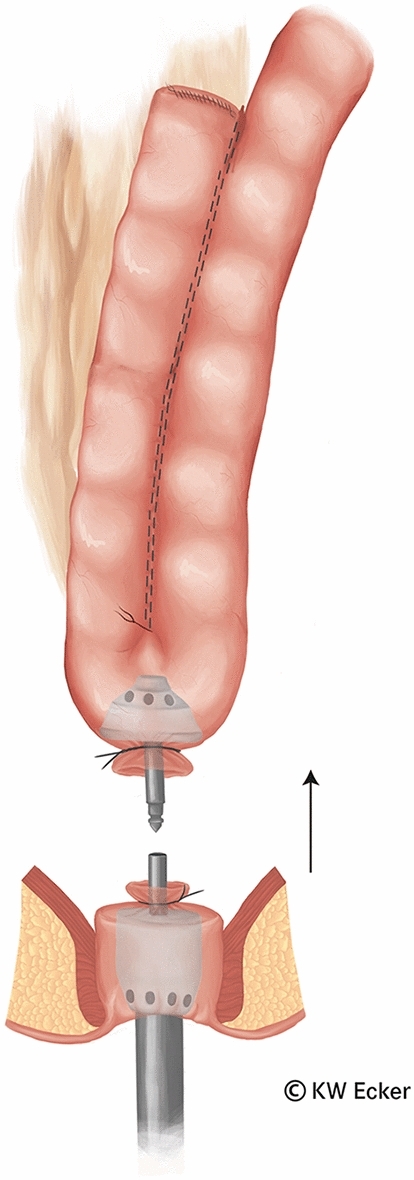
**Connecting the circular stapler.** The components of the circular stapler are now articulated together. Since the rectal stump is securely closed by the modified Asao suture, the pelvic floor can be pushed cranially by a few centimeters without risk, making it easier to reach the J-pouch with the knotted counterpressure plate

**Fig. 6:**
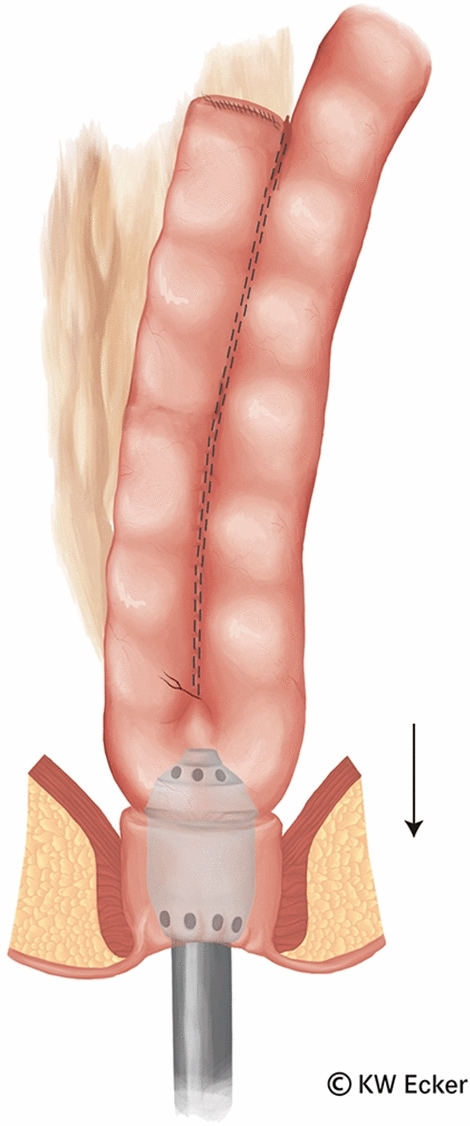
**Firing the circular stapler.** After the assembled device has been closed, the tension on the rectal stump is reduced before firing, so that no tension exists on the subsequent anastomosis

**Fig. 7:**
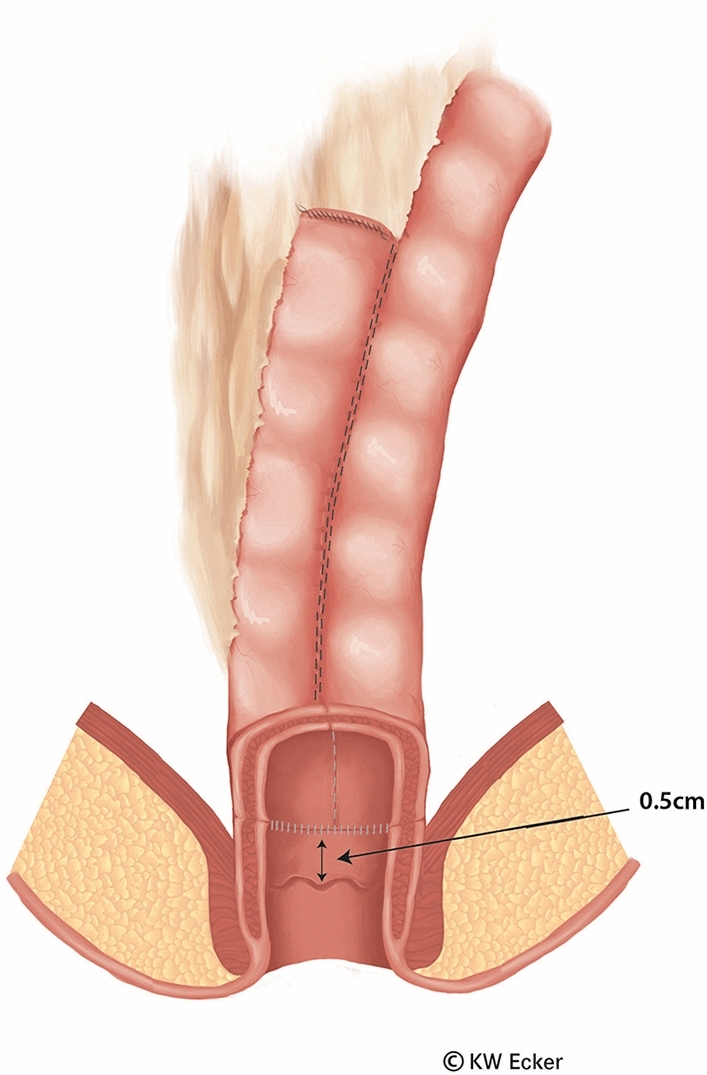
**Longitudinal section through the completed ileal pouch–anal anastomosis.** It is clear to see that this is a clean circular anastomosis without any dogears. This means that the rectal mucosa is practically totally removed, except for a margin of only a few millimeters in the region of the transition zone. The preservation of this tissue guarantees the anal sensory system, which is important for proper continence

**Fig. 8:**
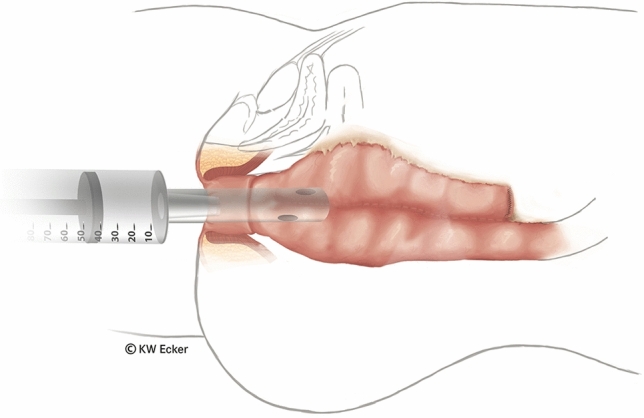
**Bubble test.** After completion of the anastomosis, a soft ileostomy catheter is inserted transanally and a bubble test is performed. If air bubbles rise in the water-filled pelvis, the anastomosis is inspected with a proctologic retractor and any leaks in the anastomosis line are sutured manually

**Fig. 9:**
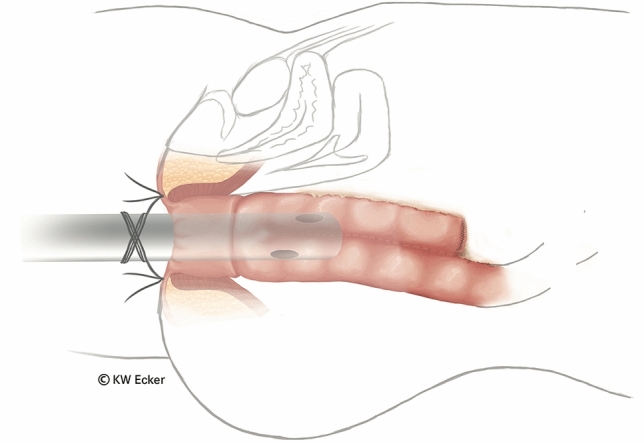
**Temporary transanal decompression.** The soft ileostomy catheter remains in place for approximately 7 days as a substitute for a defunctioning ileostomy. It is sutured to the perianal skin with two stitches to prevent dislocation. Postoperatively, the ileostomy catheter should be irrigated regularly to ensure patency. It then relieves pressure on the pouch and anastomotic sutures during the first few days and protects the vulnerable anoderm during the initial postoperative period. As the bowel content thickens, the ileostomy catheter is removed and continence training begins

Exact digital height determination, additional purse-string suture in double-stapler technique, and transanal temporary decompression by means of an ileostomy catheter are recommended to avoid all pitfalls and therefore to produce a "perfect" anastomosis in a standardized way.

## Data Availability

The data and all materials used are secured digitally by the corresponding author.

## Code availability

No code available.
